# Integration of the subarachnoid space and lymphatics: Is it time to embrace a new concept of cerebrospinal fluid absorption?

**DOI:** 10.1186/1743-8454-2-6

**Published:** 2005-09-20

**Authors:** Lena Koh, Andrei Zakharov, Miles Johnston

**Affiliations:** 1 Neuroscience Program, Department of Laboratory Medicine and Pathobiology, Sunnybrook and Women's College Health Sciences Centre, University of Toronto, 2075 Bayview Avenue, Toronto, Ontario, M4N 3M5, Canada

## Abstract

In most tissues and organs, the lymphatic circulation is responsible for the removal of interstitial protein and fluid but the parenchyma of the brain and spinal cord is devoid of lymphatic vessels. On the other hand, the literature is filled with qualitative and quantitative evidence supporting a lymphatic function in cerebrospinal fluid (CSF) absorption. The experimental data seems to warrant a re-examination of CSF dynamics and consideration of a new conceptual foundation on which to base our understanding of disorders of the CSF system. The objective of this paper is to review the key studies pertaining to the role of the lymphatic system in CSF absorption.

## Review

### Evidence for cranial CSF-lymphatic connections

The classical textbook theory assumes that the projections of the arachnoid membrane into the cranial venous sinuses represent the primary sites for CSF clearance and when absorption through these sites is blocked, this leads to disorders of the CSF system. However, this view is increasingly being contested [1-5].

Apart from one study in which Prineas [6] reported what appeared to be lymphatic vessels within the brain parenchyma of individuals with neurological disorders, it is now accepted that lymphatic vessels do not exist in the brain and spinal cord [7]. However, the literature is filled with reports of a physiological relationship between the CSF and extracranial lymph compartments.

We first learn of an affiliation between CSF and lymph through studies conducted over 100 years ago. Schwalbe demonstrated a connection between the subarachnoid space and the cervical lymphatic system in dogs and rabbits with the use of Berlin blue [8]. A similar experiment performed in dogs revealed CSF tracer in the submaxillary and cervical lymph nodes [9]. From these early studies, numerous reports solidified a link between CSF and lymph (Table 1). In general terms, these studies indicated that various tracers injected into the CSF or brain parenchyma made their way into lymphatic vessels external to the cranium and into a variety of lymph nodes in the head and neck. The cribriform plate appeared to be central to this clearance. The various tracers seemed to move from the subarachnoid space through the cribriform foramina associated with the olfactory nerves (fila olfactoria). The tracer was then observed in the lymphatic vessels in the submucosa of the olfactory and respiratory epithelium. Based on unpublished data using 20% fluorescein isothiocyanate-dextran, we found that these vessels become visible within 10 minutes after administration into the cisterna magna in sheep. As can be seen in Table 1, the association between CSF and lymph has been established in mice, rats, rabbits, cats, guinea pigs, pigs, sheep, dogs, monkeys and humans. It seems safe to assume that CSF-nasal lymphatic absorption is a characteristic feature of mammalian systems.

**Table 1:** Summary of important experiments illustrating a link between CSF and the lymphatic system

					***Site of Recovery***				
**First Author**	**Date**	**Species**	**Tracer**	**Site of ** **Injection**	***Retropharyngeal Cervical *** ***lymph nodes***	***Olfactory Nerves***	***Nasal Lymphatics***	***Nasal Interstitium***	***Spinal Nerves***

Schwalbe [8]	1869	dog, rabbit	Berlin blue	CSF	+				
Quincke [9]	1872	dog	mercuric sulfide	CSF	+				
Key & Retzius [86]	1875	human	Richardson's blue	CSF	+	+	+	+	
Goldmann [87]	1913	dog, rabbit	trypan blue	CSF	+	+			
Weed [88]	1914	cat	ferrocyanide-iron solution	CSF	+	+	+	+	
Mortensen & Sullivan [23]	1933	dog	brominized oil or thorotrast	CSF	+				
Faber [89]	1937	rabbit	X-ray medium	CSF	+	+	+	+	
Yoffey & Drinker [90]	1939	rabbit, monkey	India ink	CSF	+	+	+	+	
Brierley & Field [39]	1948	rabbit	India ink	CSF	+			+	+
Brierley [91]	1950	rabbit	India ink	CSF					+
Courtice & Simmonds [31]	1951	rabbit	blue dye plasma	CSF	+	+	+		
Simmonds [92]	1952	rabbit, cat	blood	CSF	+				
Schurr [24]	1953	dog	pantopaque	CSF	+	+	+	+	
Woollam [93]	1953	neonatal rat	colloidal carbon	CSF					+
Bowsher [94]	1957	cat	S^35 ^labeled protein	CSF					+
Svane-Knudsen [95]	1958	guinea pig	iron solution	CSF		+		+	
Di Chiro [96]	1972	dog	RISA^b^	CSF			+	+	
Potts [26]	1972	dog	radiopaque mixture	CSF		+			
Bradbury [53]	1980	sheep	RISA^b^	CSF	+				
Bradbury & Cole [54]	1980	cat, rabbit	RISA^b^	CSF	+				
Hasuo [33]	1981	dog, cat	India ink; 99 mTc-DTPA	CSF	+				
McComb [62]	1982	rabbit	RISA^b ^with dextran or dye	CSF	+	+			
Bradbury & Westrop [55]	1983	rabbit	RISA^b^	CSF	+	+			
Pile-Spellman [97]	1984	cat, rabbit	radiolabelled colloid	CSF	+	+			
Love & Leslie [63]	1984	cat	artificial CSF & dextran	CSF	+				
McComb [27]	1984	cat	RISA^b ^& Elliott's B	CSF	+	+			
Szentistvanyi [98]	1984	rat	HP^c ^& or Evan's blue albumin	PAR^a^	+	+			
Gomez [99]	1985	rabbit	HP^c ^& Evan's blue	CSF		+	+	+	
Erlich [100]	1986	rabbit	ferritin	CSF		+		+	
Leeds [101]	1989	dog	Ringer's lactate or blue dye	CSF	+				
Harling-Berg [81]	1989	rat	human serum albumin	CSF	+				
Wang & Casley-Smith [50]	1989	rat	India ink	PAR^a^	+				
McComb & Hyman [56]	1990	monkey	RISA^b^	CSF	+		+		
Yamada [102]	1991	rabbit	RISA^b^	PAR^a^	+				
Tsay [103]	1992	rabbit	saline	CSF	+				
Zhang [12]	1992	rat	India ink	CSF / PAR^a^	+		+		
Kida [11]	1993	rat	India ink	CSF	+	+/-	+	+/-	
Kida [14]	1994	rat	India ink	PAR^a^	+	+/-	+	+/-	
Botel [69]	1994	cat, rat, dog, monkey	X-ray medium	CSF	+	+		+	
Brinker [30]	1994	cat, dog, monkey	dye, dextran, X-ray medium	CSF	+	+	+	+	
Hunter [104]	1995	rabbit	nanoparticulate contrast	CSF / PAR^a^	+				
Slusarczyk [105]	1996	rat	India ink	CSF	+				
Boulton [106]	1996	sheep	RISA^b^	CSF	+				+
Miura [40]	1998	monkey	carbon particles	CSF	+				+
Boulton [58]	1999	rat	RISA^b^	CSF	+				
Silver [65]	1999	sheep	artificial CSF	CSF	+				
Bozanovic-Sosic [41]	2001	sheep	RISA^b^	CSF					+
Zakharov [16]	2003	neonatal sheep	Microfil	CSF	+	+	+	+/-	+
Vega & Jonakait [107]	2004	rat	India ink	CSF	+				
Zakharov [17]	2004	neonatal sheep	Microfil	CSF	+	+	+	+/-	
Johnston [15]	2004	sheep, pig, rabbit, rat mouse, monkey, human	Microfil	CSF	+	+	+	+/-	
Johnston [108]	2005	monkey	Microfil	CSF	+	+	+	+/-	

^a ^parenchyma

^b ^radioisotope iodinated serum albumin

^c ^horseradish peroxidase

### Nature of anatomical connections between CSF and lymphatic system

The cellular parameters associated with the physiological 'coupling' of the CSF and extracranial lymph compartments will no doubt have an influential role in determining how CSF is absorbed and by inference, also create potential pathological targets for obstruction and interference with CSF absorption. The most basic issue is whether CSF convects first into an intervening interstitial compartment (the submucosal interstitium associated with the olfactory and respiratory epithelium) or whether there is some form of direct connection between the CSF and nasal lymphatics. Table 1 highlights support for both of these propositions.

Jackson and colleagues have postulated two possible mechanisms for CSF uptake into lymphatics [10]. The first is the "open cuff model" in which the perineural sheath cells disappear distal to the cribriform plate, allowing CSF to dissipate into the interstitial space where it is absorbed by the initial lymphatics in the olfactory and respiratory submucosa. The "closed cuff model" depicts the perineural space as a cul de sac. In this case, lymphatic vessels may fuse with the perineural cells and in some way get direct access to CSF that has convected along the olfactory nerve. However, more recent data suggests a third possibility.

**Figure 1:**
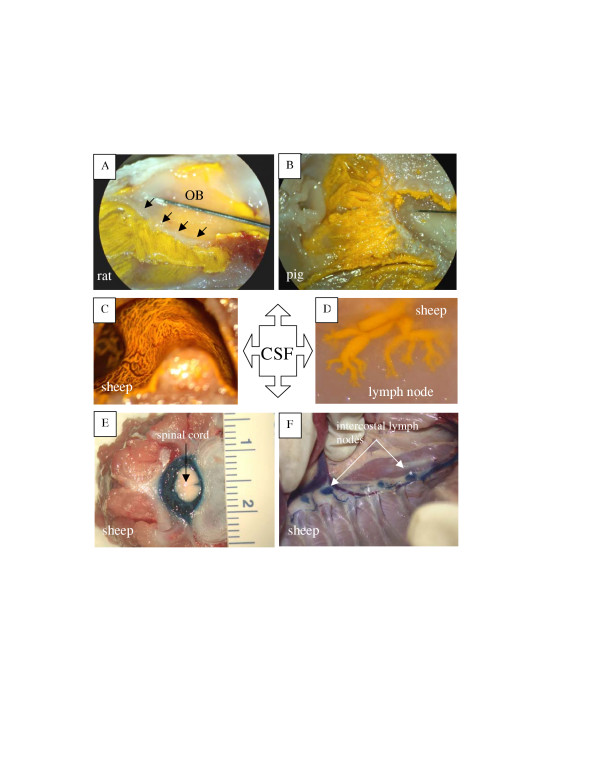
Anatomical relationships between cerebrospinal fluid and lymphatic vessels. A – Illustration of cribriform plate and lymphatic vessels in the rat. In this example, yellow Microfil has been injected into the cisterna magna. An extensive network of lymphatics filled with yellow Microfil can be observed in the olfactory submucosa. Black arrows-cribriform plate; OB – olfactory bulb. B – Lymphatics filled with yellow Microfil (injected into the cisterna magna) in the ethmoid turbinates of the pig. C – Lymphatics filled with yellow Microfil (injected into the cisterna magna) in the ethmoid turbinates of the sheep. Blood vessels (red) can be seen interspersed between the lymphatic networks. D – Lymphatics filled with yellow Microfil (injected into the cisterna magna) converge on several lymph nodes. In this example, prenodal lymphatic vessels can be observed converging onto one of the retropharyngeal nodes in sheep. E – When Evans blue dye is injected into the spinal subarachnoid space in sheep, it enters the epidural tissues around the spinal cord. F – Lymphatic vessels filled with Evans blue dye (injected into the spinal subarachnoid space) can be observed draining to the intercostal lymph nodes in sheep.

In rats, CSF may move directly from the subarachnoid space into submucosal lymphatics that emerge at the level of the cribriform plate [11-14]. This concept is supported by recent studies with Microfil, a silicon rubber injection compound. When Microfil was infused into the subarachnoid compartment of mice, rats, rabbits, sheep, pigs, monkeys and humans, it entered an extensive lymphatic network adjacent to the extracranial surface of the cribriform plate [4,15-17]. Lymphatics filled with Microfil were especially conspicuous around the olfactory nerves close to the point of exit from the cribriform plate (Figs. 1A–C). Microfil was also observed in the afferent lymphatic vessels entering into the retropharyngeal nodes (Fig. 1D). While some Microfil was scattered throughout the nasal submucosal tissues in some preparations, this pattern was the exception rather than the rule. It is possible that the high pressures required to infuse the Microfil in the *post-mortem *state could have ruptured the lymphatic vessels occasionally. Additionally, it was clear that the longer the period between death and infusion of the contrast agent, the greater the chance of Microfil being observed within the nasal interstitial space due to tissue deterioration.

It is of interest to note that some contrast agents can be taken up into lymphatic vessels readily after injection into the interstitial space *post-mortem*. Evans blue dye is an example. However, this does not seem to be true of Microfil. This silastic material was developed to outline vascular networks after injection into a vessel lumen. It is relatively viscous and is unlikely to be taken up readily from an interstitial compartment. In the Microfil studies [17], the authors failed to visualize lymphatic vessels following subcutaneous injection of Microfil. The material accumulated at the depot site but did not enter the initial lymphatics *post-mortem*. This implied that a direct connection had to exist between the CSF and lymph compartments to facilitate uptake into lymphatic vessels.

**Figure 2:**
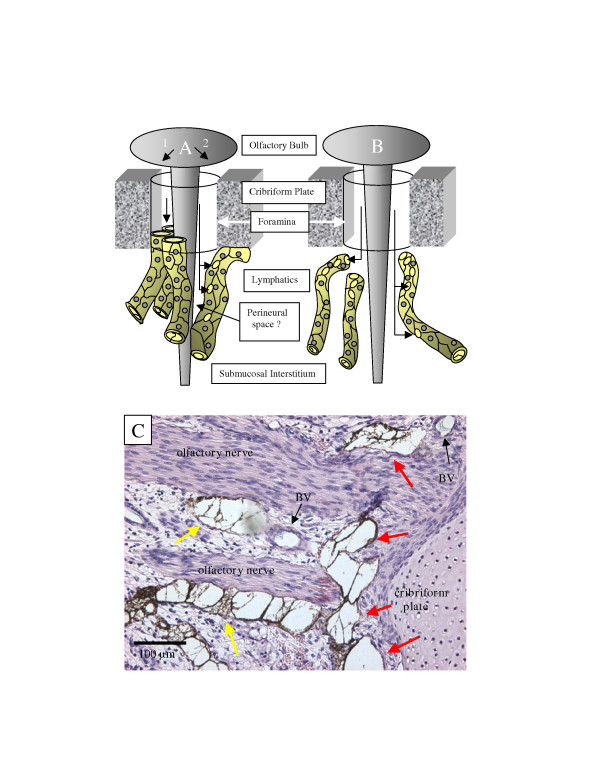
Anatomical connections between the olfactory nerve and extracranial lymphatic vessels. In schematic (A) the lymphatics are connected directly with the CSF space. In A1, the lymphatic vessels form a collar around the emerging olfactory nerve root with the lymphatic endothelium fusing to the perineural sheath of the nerve and the periosteum or dura associated with the cribriform plate. In effect this lymphatic collar provides a 'seal' that ensures that little or no CSF enters the submucosal interstitium. In A2, the lymphatics join with the cribriform plate and nerve as above but in this scenario, a collar of CSF follows the nerve some distance into the submucosa. This CSF collar is delimited by the lymphatic vessel. As in the scenario outlined in A1, no CSF is permitted to enter the interstitium. In (B), the lymphatics are not connected directly with the olfactory nerves or cribriform plate but are interspersed throughout the olfactory submucosa. In this proposal, CSF must convect first into the interstitium of the submucosa from which it is absorbed into blind ending lymphatic vessels. (C) Uptake of Microfil by lymphatic vessels adjacent to cribriform plate. This histological section was stained with hematoxylin and eosin. In this example, yellow Microfil was infused into the CSF space (appears dark brown in section) and blue Microfil was injected into the arterial circulation. Distended lymphatic vessels containing Microfil are especially prominent in the area surrounding the olfactory nerve roots as they emerge from the cribriform plate (red arrows). Lymphatics are also observed fused to the olfactory nerves at discrete locations away from the cribriform plate (yellow arrows). Microfil is not observed free within the interstitium of the submucosa. Regarding the relationship between cranial CSF and lymph, examples such as this would appear to support the schema illustrated in A. BV – blood vessels.

Histological investigation in the sheep Microfil studies showed that the lymphatic vessels fused to the sheaths of the olfactory nerves within the submucosa proximal to the cribriform plate [17]. Upon closer examination, lymphatics filled with Microfil appeared to form a collar around olfactory nerves close to the extracranial portion of the cribriform plate (Fig. 2).

Some studies have reported the existence of a perineural sheath around the olfactory nerves composed of flattened cells [18]. Whether this layer is oriented sparsely around the nerve [19] or represents a more substantial connective tissue sheath [20] is open to debate. More recently, the olfactory ensheathing cells have been identified. In mammals, these cells appear to be responsible for the regeneration of unmyelinated olfactory axons throughout life [21] and have been observed along the nerves from the olfactory mucosa to the olfactory bulbs [22]. Whatever the nature of the outer cell layer, it is evident that the lymphatic endothelial cells fuse to this tissue [17].

Direct connections between CSF and lymph would appear to make sense from a theoretical perspective. One might imagine that CSF leaks would be very common if CSF convected routinely into the nasal submucosal interstitium since the fluid would be separated from the air spaces only by a layer of olfactory or respiratory epithelium. The need for effective CSF clearance under a wide variety of intracranial pressures and the requirement to protect the brain from air-borne infection would seem to be best met by a CSF absorption system that limits CSF access to well-defined lymphatic drainage pathways rather than permit random CSF dispersion throughout the extracellular spaces of the olfactory and respiratory submucosa.

### Other CSF-lymphatic connections

The most important lymphatic CSF absorption pathway is no doubt the olfactory route leading to cervical lymphatic vessels but there are other nerves that may conduct CSF extracranially. Even though the bulk of evidence favors the olfactory nerves as facilitating CSF-lymph connections, tracers injected into the CSF system appear to exit the cranium along almost all of the cranial nerves including the trigeminal, acoustic [7], hypoglossal and vagus nerves [16].

Injection of tracer into the subarachnoid space resulted in the appearance of tracer in the optic nerve [16,23-31]. Although the eye does not appear to contain lymphatics, one report noted edema of the eye in cats following resection of the cervical lymph nodes and vessels [32]. Hasuo and colleagues proposed CSF drainage from the subarachnoid space of the optic nerve through arachnoid granulations into the orbital connective tissue from which lymphatics were believed to transfer the fluid to the cervical lymph nodes [33].

One possible location for lymphatic CSF absorption that has been ignored generally is the dura itself. In rats, lymphatics exist around the wall of the sagittal sinus, in the areas of the confluence of sinuses in proximity to the mesothelial cells of the subdural spaces and close to the vasculature of the dural tissues [34]. Lymphatic vessels have also been observed in the dura of the base of the skull of dogs [35]. ^125^I-albumin injected into the subdural space in rabbits was observed to enter plasma [36] and it seems likely that dural lymphatics contributed to this clearance. In the studies by Killer *et al*, India ink injected into the subarachnoid space of the optic nerve penetrated the arachnoid and entered the interstitial compartment and lymphatics in the dura of the nerve [37]. There is however, at least one theoretical objection to a possible role for dural lymphatics in CSF drainage. The cellular architecture and the presence of tight junctions between arachnoid cells are believed to contribute to the blood-brain/CSF barrier [38]. Without this barrier function, the extravasated fluid and solutes from the permeable dural capillaries would enter the dural interstitium and possibly gain access to CSF. However, for any dural CSF absorption to occur, presumably CSF would have to pass through the supposed barrier provided by the arachnoid membrane to enter dural tissues.

Lymphatics also appear to play a role in spinal CSF absorption. India ink infused into the ventricles or cisterna magna of rabbits has been found around emerging spinal nerve roots. The tracer passed from the subarachnoid space cul de sac into lymphatic vessels and nodes of the cervical and lumbosacral region [39]. Similarly, an accumulation of carbon particles was found in the lumbar para-aortic lymph nodes in rats following infusion of India ink into the cisterna magna [11]. In monkeys, lymphatic vessels have been observed in spinal epidural tissues [40]. Unlike the situation with olfactory nerves, there is no evidence for direct spinal CSF-lymph connections. It is clear that CSF from the spinal subarachnoid compartment must first pass into the epidural tissues from which absorption takes place into blind ending lymphatic vessels (Figs. 1E, F).

From a quantitative perspective, the drainage of CSF from the spinal cord subarachnoid space plays a role in total volumetric CSF absorption. Studies performed in sheep showed that the relative proportion of CSF absorption by the spinal compartment represents approximately 25% of total CSF clearance [41].

### Relationship between parenchymal interstitial fluid and the lymphatic circulation

The CNS has a complex extracellular space that connects with the internal (ventricular) and external (subarachnoidal) CSF through the ependymal layer and pia-mater and the Virchow-Robin spaces [42]. The Virchow-Robin spaces are extensions of the subarachnoid space (also termed perivascular spaces) that penetrate with blood vessels into the brain. Fluid within this space appears to be continuous with CSF and the parenchymal interstitial liquid [43]. Other studies have also shown a direct anatomical connection between the perivascular space of intracerebral arteries and the perivascular space of arteries in the subarachnoid space in humans and rats [43,44]. The studies of Cserr *et al *[45-47] support the concept of bulk drainage of interstitial fluid from its formation at the capillary-glial complex and its movement through the perivascular and subependymal regions into the ventricular system and subarachnoid space. Following injection of radioactive albumin into the caudate nucleus of rabbits, about 50% of the tracer cleared from the brain was accounted for by passage to lymph [48]. Additionally, Kida performed a series of studies in rats demonstrating a direct drainage of interstitial fluid and CSF into the deep cervical lymph nodes [14]. Therefore, at least a portion of parenchymal interstitial fluid drains ultimately into lymphatic vessels.

Földi developed the concept of parenchymal interstitial fluid draining into extracranial lymphatics located in the adventitia of the internal carotid artery [49]. Wang and colleagues observed that a carbon tracer injected into the cerebral hemispheres drained extracranially along the adventitia of internal carotid arteries and vertebral arteries of rats [50]. These adventitial spaces were considered to be prelymphatic, as subsequent tracer was found in the deep cervical lymph nodes.

### Quantitative evidence for volumetric CSF absorption into cervical lymphatics

It is difficult to quantify volumetric CSF absorption by lymphatics due to the complexity of the anatomical pathways involved. Some investigators have simply taken CSF tracer recovery in lymph nodes as a reflection of lymphatic function. For example, Marmarou's group measured a very low recovery in the cervical, retropharyngeal, parotid, and mandibular lymph nodes in cats 8 h after infusion of radioactive albumin into the brain [51]. However, at a given point in time, the amount of lymph within a node is very small and represents only a miniscule fraction of the mass of tracer that would have traversed the node over a given period. A more appropriate approach is to collect lymph from the cervical lymphatic vessels. For example, Boulton *et al *collected lymph from sheep cervical lymphatic vessels overtime after administration of a radioactive tracer into the cisterna magna, and found that there were measurable amounts of tracer in the lymph 1 h after injection (the rate of lymphatic CSF absorption peaked at 1.86 ml/hr, 3 h after injection) [52].

Courtice and Simmonds were among the first to quantify the absorption of a CSF dye into plasma and cervical lymph [31]. They found that on average, 4.7% of the total amount of dye injected into the CSF space was recovered in cervical lymph of cats during the 3.5–4.5 h duration of the experiment. In sheep, Bradbury and colleagues monitored cervical lymph flow for over 24 h after a single injection or continuous intraventricular infusion of I^125^-albumin. Approximately 32% of CSF was recovered in the cervical lymphatics of sheep [53]. Similar experiments were performed in the cat and rabbit (6–8 h duration), and tracer recoveries were 13% and 39%, respectively [54]. When the cribriform plate was sealed intracranially in the rabbit (with kaolin injection or with removal of the olfactory bulbs followed by application of cyanoacrylate glue to the plate), recoveries in cervical lymph dropped by approximately 90% [55]. In primates, a recovery of between 30–50% of I^125^-albumin was observed in extracranial tissue spaces and lymphatics after continuous infusion into the lateral ventricles [56].

While these studies hinted at an important role of lymphatics in CSF absorption, it was difficult to envision how protein recoveries translated into volumetric data. Additionally, a crucial element in designing an approach to quantify the lymphatic contribution to CSF absorption is the ability to correct the recovery data for errors introduced by filtration of the CSF tracer. In other words, presuming that arachnoid villi and granulations transport CSF into the plasma, the CSF in the plasma will eventually filter into the lymphatic compartment. Without correction, the cannulated lymphatic vessels might receive CSF tracer not only from the CSF compartment directly but also from re-circulated plasma tracer. This would result in an overestimation of the lymphatic contribution to CSF drainage. Similarly, the non-lymphatic contribution to CSF clearance would be underestimated if the loss of CSF tracer due to the normal filtration of proteins from the vasculature were not taken into consideration. Indeed, one study showed that the loss of tracer from sheep plasma was over 5%/h [52].

To correct the tracer recovery data for filtration errors and to permit the estimation of volumetric data from protein tracer approaches, a three-compartment mathematical model was developed and applied to sheep data. The data suggested that 40–48% of all CSF removed from the cranial compartment in adult sheep was cleared by lymphatics [52]. Additionally, plasma recoveries of a CSF tracer dropped by approximately 50% in sheep [57] and rats [58] when the cervical lymphatics were diverted or obliterated, further supporting the view that the cervical lymphatic vessels are responsible for about one-half of total CSF clearance.

While protein tracer studies have played an important role in focusing attention on lymphatic CSF absorption, perhaps the most striking data have been obtained from studies in which the cribriform plate has been sealed in sheep. In this procedure the nasal mucosa, olfactory nerves and all soft tissue on the extracranial surface of the cribriform plate were scraped away with a curette and the bone surface sealed with either bone wax or tissue glue. Sheep were challenged with constant flow or constant pressure infusions of artificial CSF into the CSF compartment before and after the extracranial side of the cribriform plate was sealed. The rate of CSF absorption was reduced significantly by this blockage and remarkably, the data suggested that the majority (> 80%) of cranial CSF absorption occurred through the cribriform plate at opening CSF pressures in adult [59] and in newborn animals [60]. When radioactive CSF protein tracers were injected into the CSF compartment of fetal sheep, the highest concentrations were measured in lymph collected from the cervical lymphatics compared with samples obtained from the thoracic duct or plasma [61]. These data suggest that lymphatics have an important role in CSF absorption before birth as well.

### Relationship between intracranial pressure and lymphatic CSF absorption

McComb and colleagues noted that an increase in intracranial pressure (ICP) in rabbits and cats resulted in greater levels of a CSF radioactive tracer in the optic nerve, olfactory bulbs, episcleral tissue, and deep cervical lymph nodes [27,62]. Hasuo observed that cervical lymph flow in dogs and cats increased 2–5 fold when ICP was raised to 30–70 cm H_2_O [33]. A temporary increase in cervical lymph flow has been observed in cats during cisternal infusions [63]. Protein concentrations declined during the experimental period due presumably to the increased amount of CSF draining via the lymph vessels.

In sheep, cervical lymphatic pressures and flow rates were closely related to ICP [64]. Silver and colleagues measured the cervical lymphatic pressure and lymph flow rates under incremental changes in ICP (10–70 cm H_2_O). At baseline CSF pressures, about 10% of the lymph in sheep cervical lymphatic vessels had its origins as CSF. As ICP was elevated, the proportion increased. At 70 cm H_2_O ICP, cervical lymph flow rates were 4 fold higher compared to baseline conditions and nearly 80% of the lymph in these ducts was estimated to originate in the CSF compartment [65].

### Implications of blockage of lymphatic CSF absorption

Edema of the brain, elevation of ICP, EEG anomalies and behavioural alterations have been demonstrated after chronic ligation of the cervical lymphatic vessels of dogs [49,66]. Similarly, removal of cervical nodes and ligation of cervical lymphatic vessels in rabbits led to cellular changes in the brain including necrotic neurons, and a dense infiltration of phagocytes [67]. Ligation of the cervical lymphatics result in edema of the brain and increased concentration of protein in cats and rabbits [32,68]. Botel and colleagues obstructed the retropharyngeal lymph nodes and vessels in cats by coagulation [69]. This group observed that CSF outflow resistance doubled, but ICP remained the same compared to control animals.

In recent studies, baseline ICP was elevated after the cribriform plate was obstructed on the nasal side [70]. Mean, diastolic, and systolic ICPs doubled when CSF absorption through the cribriform plate was prevented. An important element of the experimental design was the separation of the cranial and spinal subarachnoid compartments. With this approach, cranial CSF absorption could be assessed without the added complexities of compensatory CSF drainage mechanisms associated with the spinal cord. Therefore, with a major absorption site negated, the ability of the host to balance CSF production was impaired. In order to establish a new equilibrium condition, much higher ICPs were required.

Following bolus infusions of saline into the CSF compartment of adult sheep, obstruction of CSF absorption through the cribriform plate increased the peak ICP after infusion and augmented the time required for ICP to return to baseline [71]. Moreover, analysis of the data indicated that CSF outflow resistance was elevated significantly. Cribriform plate obstruction reduced cranial CSF absorption in adult [59] and neonatal sheep [60]. For a given ICP, CSF clearance was reduced substantially after sealing the cribriform plate. It was evident that much higher CSF pressures were required to maintain a given CSF absorption rate when CSF access to lymphatic vessels in the nasal submucosa was prevented. Additionally, obstruction of the cribriform plate also increased the concentration of the radioactive tracer in the superior sagittal sinus [3].

### Are disorders of the CSF system associated with impaired lymphatic CSF absorption?

Very little information is available on this subject but there are some interesting observations that may impact on this issue. Surgical procedures in humans that ablate the olfactory nerves do not seem to be associated regularly with any discernible problems with CSF circulation. It is plausible that CSF might be diverted to the spinal subarachnoid space (and thence, into lymphatics associated with the spinal epidural tissues) to compensate for the obstruction to absorption at the cribriform plate. Nonetheless, it is noteworthy that a study showed that 8% of patients developed hydrocephalus in the immediate postoperative period of cranial base surgery, with half of these patients also exhibiting CSF leaks [72].

Lack of development of the olfactory bulbs in humans [73] and mice [74] has also been associated with hydrocephalus. It is not clear whether the olfactory neurons are absent or defective in these examples but, if this is the case, the important lymphatic connections in the vicinity of the cribriform plate may not exist. In this regard, cranial skeletal anomalies have been associated with CSF disorders. The forkhead transcription factor *Foxc1 *mouse mutant demonstrates hydrocephalus and other defects [75]. The skeletal defects in the head are extensive with many bones being distorted or absent including those associated with the base of the skull [76]. Additionally, the nasal septum (within which a repository of lymphatics exists with known connections to the CSF compartment) is reduced in size. These alterations might affect the architecture of the cribriform plate and reduce the number of lymphatics that have access to CSF. These animals also exhibit extensive edema [76]. While no reason for the edema has yet been proposed it is of interest to note that targeted disruptions of the related *Foxc2 *gene are associated with abnormal development of lymphatic vessels [77].

The time taken for India ink to move from the CSF into the cervical lymph nodes was increased relative to controls in a model of TGFβ1 induced hydrocephalus in the mouse [78]. This suggests that the cribriform-lymphatic connection is disrupted in these animals. When bismuth (Bi) subnitrate was injected into the peritoneal cavity of mice the animals developed hydrocephalus [79]. High concentrations of Bi were present in the olfactory bulb and hypothalamus. Additionally, high Bi-levels were associated with diffusion from fenestrated blood vessels of the circumventricular organs and olfactory epithelium. Whether bismuth toxicity elicits some pathological process at the level of the olfactory-lymph connections has never been determined but seems worth investigating. Further study in these animal models may help to elucidate whether impaired lymphatic CSF absorption is linked to disorders of the CSF system.

A lymphatic-CSF relationship would also seem to have immunological implications. For example, a humoral immune response in mice was generated mainly by the deep cervical lymph nodes after injection of sheep red blood cells into various intracerebral sites [80]. In rats, infusion of human serum albumin into the cranial CSF [81] or administration of ovalbumin into the spinal subarachnoid space [82] led to antibody production by the cervical lymph nodes. Antibody titers in the peripheral circulation were reduced when cervical lymphatics were obliterated [81].

After the induction of experimental autoimmune encephalomyelitis in rats, a severe immune response was generated, resulting in cerebral lesions [83]. Removal of the deep and superficial cervical lymph nodes following induction of autoimmune encephalomyelitis reduced the severity of the pathology significantly. Therefore, the cervical lymph nodes may act to prime immune cells to target the brain. Some investigators have speculated that lymphatic drainage of brain antigens could conceivably contribute to the pathogenesis of Alzheimer's disease and multiple sclerosis [84].

## Conclusion

The tenets that form the basis of our understanding of CSF absorption do not appear to have received critical appraisal in recent years. The arachnoid projections into the cranial venous sinuses are believed to represent the primary sites for CSF absorption and current views on the pathophysiology of the CSF system have often focused on impaired CSF clearance through these elements [85]. However, this concept may be in need of revision. The possibility that CSF may drain into extracranial lymphatic vessels in significant volumes has been generally ignored even though an association between CSF and lymph has been known for over 100 years. CSF mainly flows along the extensions of the subarachnoid compartment associated primarily with olfactory nerves, convects through the cribriform plate and is absorbed ultimately by lymphatics in the nasal submucosa. It seems to be an appropriate time to create a new conceptual foundation on which to base our understanding of CSF parameters. Attention directed to lymphatic CSF absorption may reveal new insights into the cause of CSF disorders and provide novel targets for therapeutic intervention.

## Competing interests

The author(s) declare that they have no competing interests.

## Authors' contributions

LK had the primary responsibility of writing and organizing the review. AZ and MJ contributed ideas and helped in the preparation of the manuscript. All authors read and approved the final manuscript.
